# Efficient Extraction of Total Polyphenols from Apple and Investigation of Its SPF Properties

**DOI:** 10.3390/molecules27051679

**Published:** 2022-03-03

**Authors:** Ocsana Opriş, Ildiko Lung, Maria-Loredana Soran, Adina Stegarescu, Tatiana Cesco, Aliona Ghendov-Mosanu, Paula Podea, Rodica Sturza

**Affiliations:** 1 Department of Physics of Nanostructured Systems, National Institute for Research and Development of Isotopic and Molecular Technologies, 400293 Cluj-Napoca, Romania; ocsana.opris@itim-cj.ro (O.O.); ildiko.lung@itim-cj.ro (I.L.); loredana.soran@itim-cj.ro (M.-L.S.); 2 Faculty of Food Technology, Technical University of Moldova, 9/9 Studentilor St, MD-2045 Chisinau, Moldova; tatiana.cesko@saiem.utm.md (T.C.); aliona.mosanu@tpa.utm.md (A.G.-M.); rodica.sturza@chim.utm.md (R.S.); 3 Faculty of Chemistry and Chemical Engineering, Babes-Bolyai University, 11 Arany Janos Street, 400028 Cluj-Napoca, Romania; paula_mol@yahoo.com

**Keywords:** apple, sonication, reflux, SPF, emulsion

## Abstract

The purpose of this study was to evaluate the sun protection factor (SPF) of cosmetic emulsions with the addition of hydroalcoholic apple extract. First, the total polyphenolic content, the antioxidant activity and SPF properties of the extracts obtained by sonication and refluxing were evaluated. The two extraction methods were improved using the central composite design. For cosmetic emulsion that contained a different concentration of apple extract (10–40%), a SPF value between 0.51 and 0.90 was obtained. The most efficient apple extract was obtained by reflux using 50% ethanol and a 60 min extraction time. The concentrated extract was incorporated in a cosmetic emulsion whose SPF maximum was 0.90. Accordingly, due to photoprotective properties, the apple extract can be a candidate for use in cosmetic formulations.

## 1. Introduction

The number of skin cancers diagnosed annually is growing. It is well-known that this type of cancer is mainly caused by unprotected sun exposure, especially caused by ultraviolet (UV) rays [1]. Long exposure of UV radiation increases the risk of skin diseases such as cancer and skin allergic reactions, and it is necessary to find new protection methods. A common skin protection method is the use of cosmetic creams with UV protection. Chemicals identified for sun protection include PABA (para-amino benzoic acid), oxybenzone, camphor 4-methylbenzylidine, octyl methoxycinnamate, octocrylene, etc. Some of the synthetic substances with photoprotective properties used in the cosmetic industry can have limited usage due to their potential toxicity over time and due to carcinogenicity [2].

Natural substances from fruits and vegetables are recently being tested as potential sunscreen resources due to their antioxidant properties and absorption in the UV domain, and also as therapeutic products [3]. Thus, there is a considerable increase in interest in finding antioxidants from natural sources that can provide new possibilities for the treatment and prevention of diseases caused by UV radiation [4,5]. Among the antioxidants in the plant kingdom, polyphenols are among the most promising groups of compounds that have photoprotective potential [6]. The polyphenols are known for their anti-inflammatory [7,8,9], antibacterial [10,11], anticoagulant [12] and anticancer [13] activity, and contribute to preventing chronic diseases [14]. Increasing concerns in environmental protection and consumer safety have led to constant research in recent years in terms of the use of plants and fruits in cosmetics. Additionally, some compounds from plants can be used as natural surfactants because they are biodegradable. Therefore, there is a huge advantage to using them in cosmetics [15,16,17].

It is known that ultraviolet radiation causes DNA damage and it is closely correlated with mutagenesis, carcinogenesis and aging. After incubation of fibroblasts with apple extracts, Raphaelli et al. observed the DNA protection against the damage induced by UV exposure. They demonstrated that apples are the most promising in protecting fibroblast DNA against damage after UV radiation exposure [18]. Additionally, it was proven that the polyphenols, due to their antioxidant activity, are effective in topical formulations against UVB-induced skin damage to keratinocyte cells. The mechanism of action seems to be due to both direct effects on t-BHP toxicity and indirect cell-mediated effects on antioxidant defense [18,19,20]. Concerning fruit, apples are an important and unexploited source of polyphenols which are responsible for most of the antioxidant activities. Including apples in the daily diet can help to prevent degenerative disease, heart disease, diabetes and asthma [21,22,23,24,25].

In the past, the research was focused on investigating the health effects, such as the preventive effects on the body, but now, research has started to develop concerning the antioxidative side due to the important flavonoid content (e.g., flavonols, flavanols, anthocyanins, dihydrochalcones or phenolic acids) [26,27,28,29,30]. We can say that polyphenols are powerful antioxidants, and they can inhibit the reactive oxygen species’ formation as their scavengers [31,32,33,34]. They have good results as cosmetic active ingredients in cosmetic products, demonstrated by a high affinity for the reactive oxygen species (ROS), and can inhibit the formation of new ROS, by activating antioxidant enzymes, chelating pro-oxidant metal ions and neutralizing free radicals. Polyphenols can break the lipid peroxidation chain and can prevent damage of lipids, proteins and deoxyribonucleic acids in the skin, with beneficial effects on the human skin. Application of the antioxidants from natural sources, in cosmetic products, used for skin protection (from the oxidative stress, skincare after sun exposure and for prevention and anti-aging products) is very justified and became a trend in the actual dermatological deontology for skincare [16,34,35,36,37].

Chaudhary et al., in 2006, evaluated for the first time the radio-protective ability of total polyphenols extracted from the edible portion (epicarp and mesocarp) of apples by inducing oxidative damage under in vitro conditions via exposure of thymocytes to 5 Gy gamma radiations. The capacity of apples’ polyphenols to prevent DNA damage was investigated using plasmid DNA (pUC 18) on mice thymocytes by induction of single- or double-strand breaks in plasmid DNA and apples’ polyphenols. The result shows a contribution towards its overall radio-protective ability [26].

Stojiljković et al., in 2018, presented a very interesting and valuable study about in vitro and in vivo characterization of cosmetic cream with 6% aqueous extract of wild apple fruit (3.5% of alpha-hydroxyacids (AHAs) and polyphenolic compounds) [38]. Their study was performed in order to obtain a cosmetic cream with natural antioxidant active components and, at the same time, with moisturizing and bleaching properties according to the new trends of stability, safety and efficiency [34]. The conclusions presented an acceptable level of in vitro antioxidant activity, without skin irritation and with good positive results on hydrating and bleaching effects of human skin after application. These preliminary properties make this cream suitable for human use as a cosmetic product for skin damage caused by oxidative stress, along with moisturizing and bleaching of skin hyperpigmentation [38].

Some authors continued the scientific work in this area, with their target being to establish the influence of emulsifiers (conventional or biodegradable) on the properties of creams and their effects on human skin [39]. Using a conventional non-ionic mixed emulsifier, the obtained cream was stable during 180 days of storage at 22 ± 2 °C (with unchanged organoleptic properties, pH and electrical conductivity values and crystalline structures). An in vivo study revealed the absence of skin irritation after cream application under occlusion, an increase of skin moisturization and a decrease of melanin index at artificially induced skin hyperpigmented areas. The cosmetic cream containing the same concentration (6%) of standardized wild apple fruit water extract, with the same composition but stabilized by biodegradable emulsifiers, presented a better antioxidant potential and a slightly weaker moisturizing with hypopigmentation effect.

We can conclude that the regular use of these cosmetic creams may help to reduce the possibility of the harmful effects of ultraviolet radiation due to the ability to block UV radiation to prevent the negative effects of sun exposure. It is necessary to obtain a very efficient sunscreen substance for use in the cosmetic formulation with a high sun protection factor (SPF).

The aim of this paper consisted of some parameters’ optimization on obtaining an apple extract with a high content of polyphenolic compounds, antioxidant activity and sun protection factor. The apple extract with the best properties was incorporated in an emulsion as a UV filter, for which the sun-protective capacity was determined.

## 2. Results

### 2.1. Plant Extracts’ Characterization

**Figure 1:**
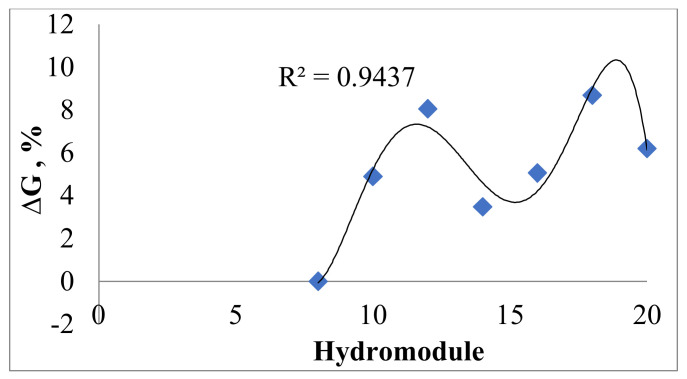
The dependence of the difference of the degree of extraction (ΔG) according to the hydromodule.

The optimum volume determined by the hydromodule required to extract the maximum amount of soluble matter from dried apples was 18 mL (Figure 1).

#### 2.1.1. Single Parameter Effect on Total Polyphenols

**Figure 2:**
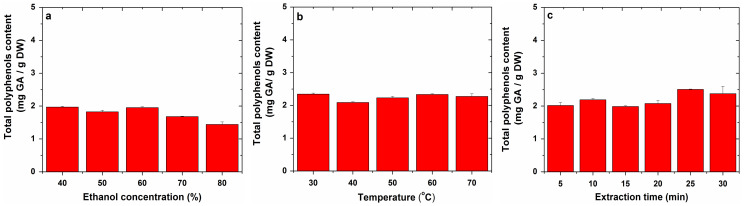
The effect of experimental parameters: ethanol concentration (**a**), temperature (**b**) and extraction time (**c**), on total phenolic content from apple extracts obtained by sonication.

**Figure 3:**
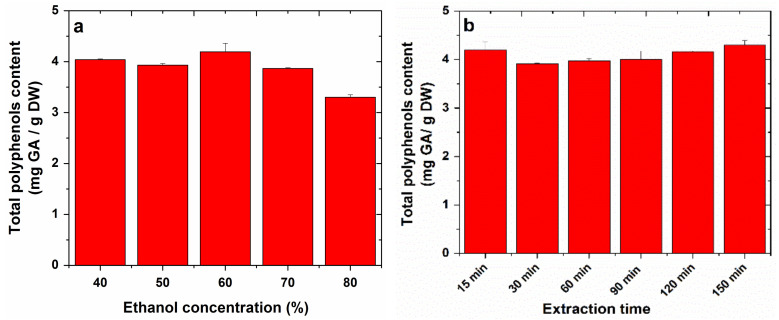
The effect of experimental parameters: ethanol concentration (**a**) and extraction time (**b**), on total phenolic content from apple extracts obtained by refluxing.

The total polyphenolic (TP) content of the analyzed extracts was calculated using the calibration curve, whose equation was y = 0.5865x + 0.0059 (R^2^ = 0.9991). The results were expressed in mg gallic acid (GA)/g dry weight (DW) of plant material, and those obtained by sonication are presented in the Figure 2, while those obtained by refluxing are shown in Figure 3.

In the case of sonication, the variation of the water–ethanol extraction solvent ratio led to the extraction of a total polyphenols content from apple, between 1.44 and 1.97 mg GA/g DW, and this content decreased with the increasing ethanol concentration. The variation of the temperature did not significantly influence the total content of polyphenols, obtaining a content between 2.09 and 2.34 mg GA/g DW. The variation of the sonication time led to a total content of polyphenols between 1.99 and 2.51 mg GA/g DW, with the highest content being obtained at a sonication time of 25 min.

In the case of reflux, testing of several water:ethanol ratios for the extraction of polyphenols from apples led to a total content of polyphenols in apples of between 0.39 and 4.19 mg GA/g DW, with the highest content being obtained with 60% ethanol. Testing for different reflux times resulted in a total polyphenol content of between 3.92 and 4.30 mg GA/g DW, which increased slightly with the increasing reflux time.

#### 2.1.2. Single Parameter Effect on Antioxidant Activity

**Figure 4:**
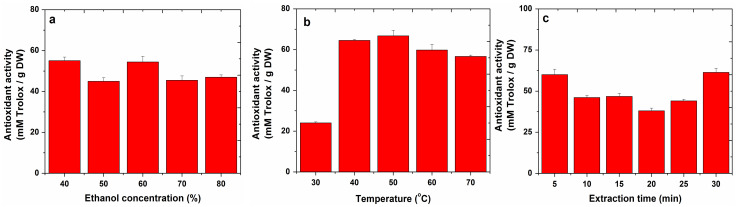
DPPH radical scavenging activity of apple extracts obtained by sonication in different conditions: ethanol concentration (**a**), temperature (**b**) and extraction time (**c**).

**Figure 5:**
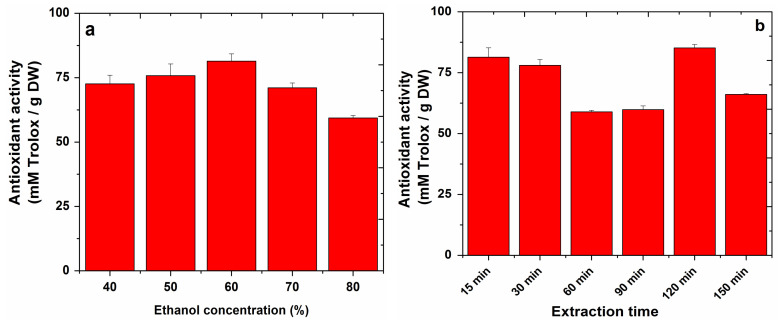
DPPH radical scavenging activity of apple extracts obtained by refluxing in different conditions: ethanol concentration (**a**) and extraction time (**b**).

The antioxidant capacity of the apple extracts was calculated from the calibration curve: y = 0.2003x + 0.0119 (R^2^ = 0.9990), and estimated in mM Trolox/g DW. The results obtained by sonication and refluxing are presented in Figure 4 and Figure 5.

Testing sonication for apple extraction, the highest antioxidant capacity of 55.09 mM Trolox/g DW was obtained using an ethanol concentration of 40% as the extraction solvent, and the highest antioxidant capacity of 81.37 mM Trolox/g DW was obtained by testing refluxing for apple extraction using a 60% ethanol concentration.

In the case of sonication, the temperature at which the highest antioxidant capacity was obtained (66.77 mM Trolox/g DW) for apple extracts was 50 °C.

Assessing the influence of extraction time on the antioxidant capacity of apple extracts, in the case of sonication, an antioxidant capacity of 61.38 mM Trolox/g DW was obtained in a time of 30 min, while in the case of refluxing, an antioxidant capacity of 85.19 mM Trolox/g DW was obtained in 120 min.

The antioxidant activity of the extract is given by the polyphenols, including phenolic acids, flavonoids, tannins, etc. [40]. Quercetin and its glycosides (isoquercitrin, rutin) are the most widespread polyphenols [39]. These polyphenols and several other phenolic compounds have DPPH scavenging activity [41].

#### 2.1.3. Single Parameter Effect on SPF

**Figure 6:**
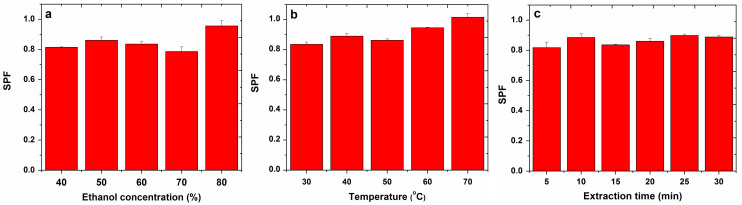
Influence of ethanol concentration (**a**), temperature (**b**) and extraction time (**c**) on SPF determined for apple extracts, obtained by sonication.

**Figure 7:**
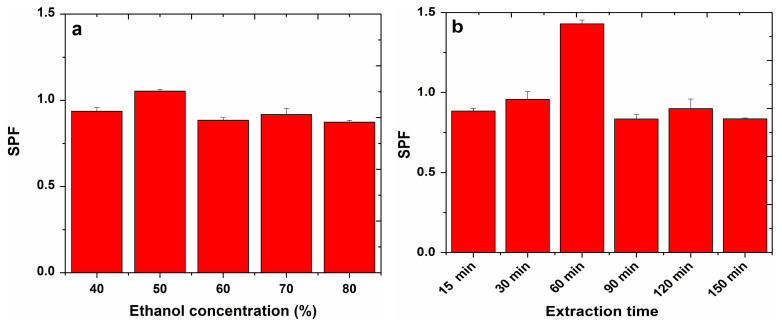
Influence of ethanol concentration (**a**) and extraction time (**b**) on SPF determined for apple extracts, obtained by refluxing.

The results obtained for the SPF determination from apple extracts by sonication and refluxing are presented in Figure 6 and Figure 7.

Assessing the influence of ethanol concentration, in the case of sonication, the highest SPF (0.96) for apple extracts was obtained with 80% ethanol, while in the case of refluxing, the highest SPF (1.05) was obtained with a 50% ethanol concentration.

In the case of sonication, the temperature at which the highest SPF (1.02) was obtained for apple extracts was 70 °C.

Evaluating the influence of the extraction time on the SPF values of the apple extracts, in the case of sonication, an SPF of 0.90 was obtained at 25 min, and in the case of refluxing, an SPF of 1.43 was obtained at 60 min.

Several studies have proven that the photoprotective capacity of vegetable extracts is due to the presence of polyphenols [42]. Among the polyphenols with photoprotective capacity would be rutin, quercetin and kaempferol [42,43,44].

The antioxidant activity and SPF are not closely correlated with polyphenol contents because these are also influenced by other compounds that can be extracted together with polyphenols.

### 2.2. Improving Apple Extracts

The central composite design (CCD) experimental design was used to improve the extraction of polyphenols from apple using sonication and refluxing. Thus, 20 experimental variants were generated and tested in the case of sonication and 13 experimental variants in the case of refluxing. The experimental data obtained for both extraction methods are presented in Table 1 and Table 2.

Following the analysis of the extracts, the highest TP content in apples (3.82 mg GA/g DW) using sonication was obtained with experimental variant No. 18 (extraction solvent: 50% ethanol, temperature: 76.33 °C, sonication time: 10 min). The highest antioxidant capacity (74.41 mM Trolox/g DW) was obtained with experimental variant No. 2 (extraction solvent: 50% ethanol, temperature: 60 °C, sonication time: 10 min). In the case of SPF of apple extracts, the highest value (0.77) was obtained with experimental variant No. 9 (extraction solvent: 40% ethanol, temperature: 70 °C, sonication time: 5 min).

Following the extracts’ analysis, the highest TP content (5.02 mg GA/g DW) using refluxing was obtained with experimental variant No. 3 (extraction solvent: 40% ethanol, refluxing time: 70 min). The highest antioxidant capacity (75.76 mM Trolox/g DW) was obtained with experimental variant No. 8 (extraction solvent: 74.14% ethanol, reflux time: 50 min). As far as SPF is concerned, the highest quantity (1.01) was obtained with experimental variants No. 10, 12 and 13 (extraction solvent: 50% ethanol, reflux time: 60 min).

### 2.3. Emulsion Characterization

After improvement, the best apple extract, with the best properties and that was obtained in a short time, was chosen and was incorporated into a cosmetic emulsion. The best extract was obtained by reflux, with extraction solvent: 50% ethanol and reflux time: 60 min.

**Figure 8:**
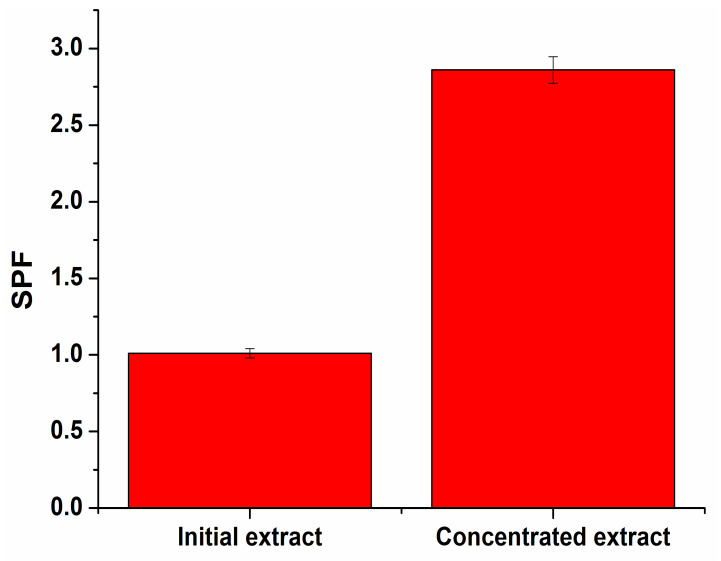
The extract’s SPF value compared to the concentrated one.

The obtained extract was concentrated by a rotary evaporator for increasing SPF (Figure 8).

**Figure 9:**
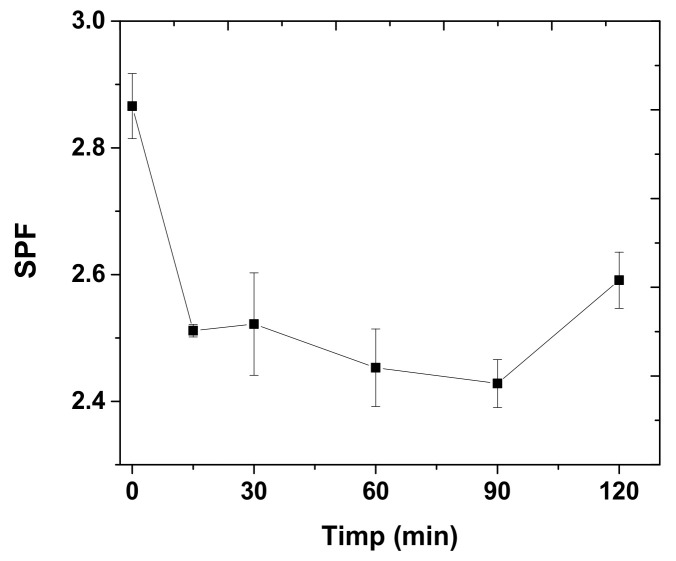
SPF of apple extract as a function of irradiation time.

The photostability of the apple extract was monitored by subjecting it to UV light using a 230 V/36 W UVA lamp and testing different exposure times (Figure 9).

Compared to the initial SPF value of apple extracts, after exposure to UV light, SPF values decreased by up to 15%.

Cosmetic emulsions with different contents of apple extract were investigated and the SPF was determined (Table 3).

Depending on the amount of extract added, the SPF values of the obtained cosmetic emulsions were different. Thus, the highest SPF was obtained for the emulsion that contained 40% apple extract.

## 3. Materials and Methods

### 3.1. Polyphenolic Extract Preparation

#### 3.1.1. Preparation of Vegetable Material

Dehydrated apples used to obtain polyphenolic extracts were purchased from the market. The dried apple slices (BDM Processing Impex SRL, Romania) were obtained by dehydrating fresh apples, without the addition of sugar or other artificial flavors. These slices were ground before being subjected to the extraction process.

#### 3.1.2. Determination of Hydromodule

Determination of each hydromodule (8, 10, 12, 14, 16, 18, 20) was performed by mixing 3 g of ground apples with the corresponding volume of double-distilled water. The resulted mixture was left to rest. Then, the percentage of dry substance was read at the refractometer until it remained constant.

#### 3.1.3. Obtaining Alcoholic Extract

Improvement of apple extracts was achieved by testing two extraction techniques (sonication and reflux) and several experimental parameters (water:ethanol ratio, temperature and extraction time).

The influence of the temperature (30–70 °C), extraction time (5–30 min) and the water:ethanol ratio (60:40–20:80, *v*/*v*) on the total apple polyphenol content was assessed.

Over 1 g of dried and ground apples, 18 mL of solvent (the ratios of water:ethanol (60:40–20:80, *v*/*v*)) was added and the mixture was sonicated (Transsonic T 310 at 35 kHz and installed power of 95 W) at different temperatures for a certain time. Another 1 g of dried and ground apples with 18 mL of solvent (the same ratios of water:ethanol as above) was added and subjected to reflux (66 °C) for a certain time. At the end, the samples were centrifuged at 7000 rpm for 10 min, after which the extracts were separated and stored in the refrigerator until analysis.

In addition to improving with the one-variable-at-a-time approach, the CCD experimental design was used to make the extraction parameters more efficient in the case of both sonication and reflux (Table 4 and Table 5).

### 3.2. Characterization of the Obtained Extracts

#### 3.2.1. Total Phenolic Content

The TP content of the extracts obtained from apples was determined by the Folin–Ciocâlteu method [45]. For this purpose, over 5 mL of double-distilled water, 100 µL of extract and 0.5 mL of Folin–Ciocâlteu reagent were added. The mixture was stirred and allowed to stand for 3 min, then 1.5 mL of Na_2_CO_3_ (5 g/L) and double-distilled water were added, up to 10 mL. The samples thus obtained were kept at 50 °C (in a water bath) for 16 min, after which they were cooled to room temperature. Their absorbance was read at 765 nm relative to the control sample (double-distilled water) using a T80 spectrometer (PG Instruments, UK). In order to determine the total amount of polyphenols, the calibration curve for GA was drawn for the range 0.002–0.8 mg/mL. The solutions in this concentration range were prepared from a standard 1 mg/mL GA solution.

#### 3.2.2. Antioxidant Capacity

The method of Brand-Williams et al. [46] was slightly modified and was used to determine the antioxidant activity of apple extracts. Thus, 10 μL of extract was added to a 3.9 mL solution (0.0025 g/100 mL methanol) of DPPH radical (2.2′-diphenyl-picrylhydra-zyl, Merck, Germany). The absorbance of the mixture, after standing in the dark for 10 min, was read at 515 nm before the control sample (10 μL extract added to 3.9 mL). The antioxidant activity of apple extracts was determined using the calibration curve drawn for Trolox ((±) 6-Hydroxy-2,5,7,8-tetramethylchromane-2-carboxylic acid, Sigma-Aldrich, Germany) for the concentration range 0–400 µM (obtained by successive dilutions).

#### 3.2.3. Determination of the SPF of Extracts

The SPF determination in vitro was performed following the Mansur method [47,48]. Thus, for the solutions with a concentration of 1% apple extract in ultrapure water, the absorbances were recorded in the range of 200–450 nm, at intervals of 5 nm, using ethanol as a control sample.

### 3.3. Incorporation of the Apple Extract in a Sunscreen Formulation

The ingredients used to obtain the emulsion and their roles are presented in Table 6.

Obtaining emulsions consists of three steps. In the first stage, the oily phase of the emulsion was obtained. For this, the fats (stearin, lanolin, glycerin, isopropyl myristate, cetyl alcohol, glyceryl stearate, medole oil, Labs-A) were melted in a water bath and heated to 75 °C. They were constantly mixed for homogenization and to avoid their degradation. In the second stage, the aqueous phase was obtained as follows: Triethanolamine, microcare, EDTA and Sabowax EL-H-KOL were dissolved in water heated to 80 °C. The aqueous phase was added in a thin thread over the oily phase. The above was mixed for 15 min in the water bath, and then continued at room temperature, thus ensuring a slow cooling. In the third stage, the plant extract (in different concentrations: 10–40%) and the perfume composition were added to the mixture when its temperature reached 40 °C. Mixing was continued until complete cooling and a once a homogeneous emulsion was obtained.

### 3.4. Sunscreen Characterization

In order to determine the SPF of the prepared emulsions, an extraction of 250 mg of emulsion was performed, to which 19 mL of ethanol was added. The mixture was sonicated at room temperature for 10 min. Over this mixture, another 6 mL of ethanol was added, after which the obtained solution was filtered, with the first 5 mL of filtrate being discarded. The absorbance of the filtrate was recorded in the range of 200–450 nm.

### 3.5. Data Analysis

Data obtained in this study were presented as mean values ± the standard error of the mean, calculated from three parallel experiments. Microsoft Office Excel 2007 (Microsoft, USA) was used to perform the calculations, while ORIGIN 9 (Origin Lab Corporation, Northampton, MA, USA) was used to graphically represent the results. CCD, using Minitab 17 (Minitab Ltd., Coventry, UK), was used to improve the extraction parameters.

## 4. Conclusions

Most synthetic sunscreens produce unwanted effects, so there is a need for UV filters of natural origin. Plants are preferred because they are rich in natural and safer compounds, which are able to absorb UV radiation and have photoprotective activity. Among the natural compounds, polyphenols have been widely explored for their radiation-absorbing properties. Numerous studies have also shown that these natural compounds have improved the SPF values and the effectiveness of commercial UV filters.

Thus, in this study, the SPF values of hydroalcoholic extracts obtained from apples by sonication and reflux were evaluated. Additionally, the total amount of polyphenols and the antioxidant activity for the obtained extracts were determined. Comparing the two extraction methods, it was found that by refluxing, the highest amounts of polyphenols (5.02 mg GA/g DW) and the highest antioxidant activity (75.76 mM Trolox/g DW) were obtained. For the efficiency, it was found that the highest value of SPF (1.01) was obtained by refluxing. The optimal parameters for obtaining the apple extract consisted of 50% ethanol as the extraction solvent and a 60 min reflux time.

For this apple extract, it was found that it is stable, because after exposure to UV light for 120 min, the SPF value decreased by only 15% of the initial value.

After determining the efficient apple extract concentration, it was incorporated in a cosmetic emulsion without other commercial UV filters. An SPF value of 0.51–0.90 was obtained for this emulsion, depending on the amount of extract added. The highest SPF value was obtained for the emulsion with 40% (volume percent) apple extract. Therefore, apple extract could be used in cosmetic formulations because it is safe and can be used as an active ingredient in sunscreens.

## Figures and Tables

**Table 1:** Experimental values of TP, DPPH and SPF for apple extracts obtained by sonication and measured at design points.

Design Point	Ethanol Concentration (%)	Temperature (°C)	Extraction Time (min)	TP (mg GA/g DW)	DPPH (mM Trolox/g DW)	SPF
1	40.00	70.00	15.00	3.58 ± 0.015	61.15 ± 1.91	0.76 ± 0.0005
2	50.00	60.00	10.00	3.59 ± 0.031	74.41 ± 2.38	0.74 ± 0.010
3	60.00	50.00	15.00	3.46 ± 0.169	71.04 ± 5.72	0.72 ± 0.007
4	50.00	60.00	10.00	3.46 ± 0.169	71.04 ± 5.72	0.72 ± 0.007
5	40.00	50.00	15.00	3.49 ± 0.077	68.79 ± 2.22	0.69 ± 0.015
6	40.00	50.00	5.00	3.56 ± 0.153	61.83 ± 2.07	0.67 ± 0.003
7	60.00	70.00	15.00	3.52 ± 0.077	73.96 ± 13.82	0.70 ± 0.0008
8	50.00	60.00	10.00	3.46 ± 0.169	71.04 ± 5.72	0.72 ± 0.007
9	40.00	70.00	5.00	3.47 ± 0.031	52.62 ± 3.49	0.77 ± 0.031
10	60.00	70.00	5.00	3.55 ± 0.046	57.11 ± 4.77	0.73 ± 0.012
11	60.00	50.00	5.00	3.44 ± 0.031	54.19 ± 2.07	0.73 ± 0.015
12	50.00	60.00	10.00	3.46 ± 0.169	71.04 ± 5.72	0.72 ± 0.007
13	50.00	60.00	18.41	3.58 ± 0.107	62.28 ± 0.16	0.71 ± 0.017
14	50.00	60.00	1.59	3.36 ± 0.077	57.56 ± 1.27	0.67 ± 0.020
15	66.82	60.00	10.00	3.78 ± 0.308	59.36 ± 2.86	0.73 ± 0.020
16	50.00	43.18	10.00	3.53 ± 0.061	56.88 ± 1.11	0.75 ± 0.015
17	33.18	60.00	10.00	3.64 ± 0.169	66.99 ± 7.94	0.72 ± 0.010
18	50.00	76.82	10.00	3.82 ± 0.107	71.26 ± 5.88	0.70 ± 0.005
19	50.00	60.00	10.00	3.46 ± 0.169	71.04 ± 5.72	0.72 ± 0.007
20	50.00	60.00	10.00	3.46 ± 0.169	71.04 ± 5.729	0.72 ± 0.007

**Table 2:** Experimental values of TP, DPPH and SPF for apple extracts obtained by refluxing and measured at design points.

Design Point	Ethanol Concentration (%)	Extraction Time (min)	TP (mg GA/g DW)	DPPH (mM Trolox/g DW)	SPF
1	35.86	60.00	4.36 ± 0.061	57.11 ± 0.32	0.87 ± 0.001
2	40.00	50.00	4.81 ± 0.077	68.34 ± 5.08	0.98 ± 0.006
3	60.00	70.00	5.02 ± 0.046	60.25 ± 2.22	0.92 ± 0.009
4	60.00	50.00	4.12 ± 0.092	59.81 ± 9.53	1.00 ± 0.270
5	50.00	60.00	4.28 ± 0.015	56.21 ± 3.18	1.00 ± 0.017
6	50.00	60.00	4.27 ± 0.015	56.21 ± 3.18	1.00 ± 0.017
7	64.14	60.00	4.25 ± 0.123	48.35 ± 2.38	0.99 ± 0.065
8	50.00	74.14	4.12 ± 0.046	75.76 ± 7.47	0.94 ± 0.014
9	50.00	45.86	4.28 ± 0.031	54.64 ± 3.34	0.95 ± 0.001
10	50.00	60.00	4.48 ± 0.015	56.21 ± 3.18	1.01 ± 0.017
11	40.00	70.00	4.28 ± 0.123	60.25 ± 0.95	0.93 ± 0.017
12	50.00	60.00	4.28 ± 0.015	56.21 ± 3.18	1.01 ± 0.017
13	50.00	60.00	4.28 ± 0.015	56.21 ± 3.18	1.01 ± 0.017

**Table 3:** SPF ± SE values obtained for cosmetic creams with concentrated plant extracts.

Extract Concentration in Cosmetic Emulsion (%)	SPF ± SE
10	0.51 ± 0.007
20	0.64 ± 0.001
30	0.80 ± 0.003
40	0.90 ± 0.001

**Table 4:** Areas of variation of the experimental conditions necessary to improve the extraction of polyphenols from apples using sonication.

Variable	(−1.68)	(−1)	(0)	(1)	(1.68)
Ethanol concentration (%)	33.2	40	50	60	66.8
Temperature (°C)	43.2	50	60	70	76.8
Extraction time (min)	1.6	5	10	15	23.4

**Table 5:** Areas of variation of the experimental conditions necessary to improve the extraction of polyphenols from apples using refluxing.

Variable	(−1.41)	(−1)	(0)	(1)	(1.41)
Ethanol concentration (%)	35.9	40	50	60	64.1
Extraction time (min)	45.9	50	60	70	74.1

**Table 6:** The composition of the emulsion.

Ingredient	Quantity (g)	The Role of the Ingredient
Purified water	35.3–65.30	Base
Stearin	3.0	Consistent emollient factor
Glyceryl stearate	2.0	Emulsifier
Lanolin	1.0	Emollient
Glycerin	1.0	Consistency factor
Medole oil	4.0	Lubricating emollient
Isopropyl myristate	5.0	Emollient
Cetyl alcohol	1.50	Consistency factor
Sabowax EL-H-KOL	5.0	Emulsifier
Triethanolamine	0.800	Neutralizing agent
Microcare	0.700	Preservative
EDTA	0.100	Complexing agent
Labs-A	0.100	Emulsifier
Apple extract	10–40	Active principle
Perfume composition	0.300	Perfume
